# Effect of screening by clinical breast examination on breast cancer incidence and mortality after 20 years: prospective, cluster randomised controlled trial in Mumbai

**DOI:** 10.1136/bmj.n738

**Published:** 2021-03-19

In this paper by Mittra and colleagues (*BMJ* 2021;372:n256, doi:10.1136/bmj.n256, published 24 February 2021), coauthor Surendra S Shastri is also affiliated at the Department of Health Disparities Research, Division of Cancer Prevention and Population Sciences, University of Texas M D Anderson Cancer Center, Houston TX, USA. The paper will be updated with the additional address in due course.

